# Coping with Shifting Nest Predation Refuges by European Reed Warblers *Acrocephalus scirpaceus*

**DOI:** 10.1371/journal.pone.0115456

**Published:** 2014-12-18

**Authors:** Lucyna Halupka, Konrad Halupka, Ewelina Klimczuk, Hanna Sztwiertnia

**Affiliations:** 1 Ornithological Station, University of Wroclaw, Wroclaw, Poland; 2 Department of Behavioural Ecology, University of Wroclaw, Wroclaw, Poland; 3 Museum of Natural History, University of Wroclaw, Wroclaw, Poland; Hungarian Academy of Sciences, Hungary

## Abstract

Predation, the most important source of nest mortality in altricial birds, has been a subject of numerous studies during past decades. However, the temporal dynamics between changing predation pressures and parental responses remain poorly understood. We analysed characteristics of 524 nests of European reed warblers monitored during six consecutive breeding seasons in the same area, and found some support for the shifting nest predation refuge hypothesis. Nest site characteristics were correlated with nest fate, but a nest with the same nest-site attributes could be relatively safe in one season and vulnerable to predation in another. Thus nest predation refuges were ephemeral and there was no between-season consistency in nest predation patterns. Reed warblers that lost their first nests in a given season did not disperse farther for the subsequent reproductive attempt, compared to successful individuals, but they introduced more changes to their second nest sites. In subsequent nests, predation risk remained constant for birds that changed nest-site characteristics, but increased for those that did not. At the between-season temporal scale, individual birds did not perform better with age in terms of reducing nest predation risk. We conclude that the experience acquired in previous years may not be useful, given that nest predation refuges are not stable.

## Introduction

Nest predation is the most important source of mortality in altricial birds [1]. In many species and habitats, however, nest predation risk varies predictably with nest-site characteristics, thus predation refuges, defined as places where predation is lower than elsewhere, may exist [2], [3], [4], [5], [6], [7], [8], [9], [10], [11]. An obvious question arises why do only some individuals nest in less vulnerable sites? Hypotheses include that nesting in refuges may be costly due to strong competition for safe locations [12], [13] or greater energetic expenditure due to adverse microclimate [14], [15]. Some evidence also suggests that knowledge about refuges is not hard-wired but must be acquired through trial-and-error learning [16]. Such a process may be modelled as a multi-armed bandit game [17], [18], in which a simple rule of thumb "repeat your choice if successful, otherwise switch to another option" (also known as "play the winner, switch from the loser" or "win-stay, lose-shift") approximates an optimal strategy. The results of some studies, in which choices of individual birds in subsequent breeding episodes were followed, suggest that parents used this rule to select nest sites. Thus, pinion jays *Gymnorhinus cyanocephalus*, after several trials, found the optimal distance of the nest to the tree top [19]. Recently, a similar self-improvement correlated with age of the individual has been found in Daito white-eyes *Zosterops japonicus* [20]. A simple case of the "win-stay, lose-shift" tactic was also described in the context of decision-making involving reuse of the old nest [21]. Finally, some researchers suggest that a similar tactic determines habitat selection: in comparison with adults from successful nests, unsuccessful parents tend to disperse more between breeding attempts in the same season, and also between years [22], [23], [24], [25].

The "win-stay, lose-shift" rule might be a good tactic in stable environments. If a given set of nest-site/patch characteristics always produces the same probability of successes/failures, this then creates an opportunity of gradual improvement in reproductive performance. Many studies demonstrate, however, that nest predation refuges are not stable in space-time and instead their locations may change [4], [26], [27], [28], [29], [30], [31], [32]. The most likely mechanism behind this depends on a relative abundance of various species in the guild of nest predators. Research using video techniques finds evidence that different predators specialize in different microniches [9], [32], [33], [34], [35], [36]. The behaviour of predators is, however, plastic and they can learn to find prey more effectively when a specific nest type becomes common [10], [37], [38], [39]. Weidinger [36] suggests that an additional important source of spatial and temporal variation in nest predation risk is connected to learned competences of individual predators. Thus woodpeckers are widely distributed potential nest predators, but they only pose a serious threat locally, if some particular individuals learn how to search and plunder nests and specialize to exploit this niche. This could even create nest predation hot spots where the risk is elevated.

Overall, it might be proposed that in the ‘shifting refuges' scenario the proportion of successful nests is a product of a complex game between predators and parent birds, played in a changing environment and involving parties with participants varying in experiences, competences and habits. Such a game can locally produce refuges that may be described by statistical rules explaining nest survival. Given the volatility of the whole system, however, their permanence might be rather low. Here, we test the shifting refuges hypothesis using data on nest success in European reed warblers breeding in the same reedbed in several consecutive breeding seasons. In particular, we test whether the pattern of nest predation varies within a breeding season, as well as between breeding seasons (a nest with certain characteristics could be safe in one season and vulnerable in another). The alternative hypothesis is that the pattern of nest predation is consistent, so nests possessing certain characteristics survive always better than other nests. We also test if parent birds can use experience to update the characteristics of subsequent nest sites. In particular, we test whether they apply the "win-stay, lose-shift" tactic to increase breeding success and whether their reproductive competence improves with age, as a result of learning.

## Methods

### Study area and species

In 2006–2011 we studied a population of European reed warblers breeding on a 4 ha plot situated within a reedbed on a 164 ha fish-pond in the nature reserve Stawy Milickie (SW Poland). The plot (centre at 51.5385°N, 17.3390°E) has roughly rectangular shape (approximately 150×270 m) and consists of patches of vegetation separated by numerous bays and narrow paths. The dominant plant species is the common reed *Phragmites australis*, in some places accompanied by bittersweets *Solanum dulcamara* and fringed by cattails *Typha angustifolia*. The plot is surrounded by a dike overgrown with trees and bushes of willows and alders from the south, and an open water of the pond from the north. Breeding habitat changes markedly throughout the breeding season of reed warblers. At the time of the first-egg laying new reeds are relatively short (median 126 cm above water level) and sparse while in late season they can exceed 4 m. Water level also fluctuates significantly; during hot dry periods water in the pond can drop by more than 40 cm.

The breeding season of the reed warbler starts in May and continues for three months. Only females build nests, but both sexes incubate eggs and feed the young. The nest has a form of a cup and the female attaches it to several stems of reeds. Clutch size varies between 3 and 5 eggs (median equals 4) and the female lays one egg per day. Eggs are incubated for 11 days after clutch completion, and the young fledge when they are 10–13 days old (median 12 days). The density of breeding pairs changes over the season, but the annual maximums range between 13 to 18 pairs/ha. For other details about study site and population see Halupka et al. [40].

Potential nest predators include the western marsh harrier *Circus aeruginosus*, hooded crow *Corvus corone*, magpie *Pica pica*, bittern *Botaurus stellaris*, little bittern *Ixobrychus minutus*, several species of mustelids (*Martes sp.*, *Mustela sp*., *Neovison vison*, *Lutra lutra*), muskrat *Ondatra zibethicus*, water snake *Natrix natrix* and rodents (*Arvicola terrestris, Micromys minutus*). The reedbed is also inhabited by numerous bird species which might be opportunistic nest predators, e.g.coot *Fulica atra*, water rail *Rallus aquaticus*, moorhen *Gallinula chloropus,* and spotted crake *Porzana porzana*. Reed warblers are sometimes hosts of the common cuckoo *Cuculus canorus* that can also be a locally important nest predator [41], [42]. However, in our population cuckoo parasitism was low:1.5–7% (med  = 2.6%), and parasitised nests were found usually within 30 m of the dike overgrown with trees serving as vantage points [43], [44], while predation occurred in all parts of the reedbed. Additionally, the proportion of parasitised nests and nests destroyed by predators did not correlate, indirectly suggesting that the cuckoo is not an important predator in our study area. For example, in 2007, when the level of cuckoo parasitism was the highest, we recorded relatively low level of nest predation on the whole study plot, and very low (11.1%) in the part of the reedbed penetrated by cuckoos.

### Field procedures

We attempted to find all nests on the study plot, although it is possible that a few remained undetected. We searched for nests by observing nest-building, mate-guarding and parental activities from two 6 m high wooden towers or 3 m portable aluminium ladders. Throughout the entire season we captured (mist-nets) adult birds and ringed them with unique combinations of colours. In 2006–2011 a total of 373 males and 305 females were ringed. Parental birds were identified at nests by direct observation during nest-building or by video-recording of nests. Of all the birds ringed, 18% of females and 28% of males returned to the study plot during successive breeding seasons.

Altogether we found 532 nests with eggs or nestlings. Of those, 524 (99%) were included in the final analysis. We decided to exclude two nests that, when found, already had a cuckoo egg, two nests for which we could not estimate the date of clutch initiation (they were found at the incubation stage and depredated before hatching), and four nests where characteristics of nest-sites were not measured. Most nests (85% of 524) were found at the building or egg laying stages. When a nest with a complete clutch was found, we monitored it until hatching day and then backdated to estimate the clutch initiation date (assuming one egg laid per day and an incubation period of 11 days after clutch completion). If a newly found nest contained nestlings, their age was determined based on comparisons with known age-associated features (body weight, feather development, degree of eye opening, etc.). Nests were checked usually every second day or, during egg-laying and before the expected hatching and fledging dates, daily. Therefore, the day of fledging or the day of nest destruction could usually be determined accurately; in a few uncertain cases we used the mid-point assumption. Nests were classified as successful when at least one young left the nest. We assumed that if a nest contained young on day 11, it was successful, following Honza et al. [45]. The additional condition to classify a nest as successful was asynchronous nest leaving (following asynchronous hatching pattern). In our experience the synchronous disappearance of nestlings (even if they were potentially ready to leave the nest) was associated with nest predation.

Measurements of characteristics of the nest site were taken on the day following the date of the last egg being laid, always by the same person (LH). When a nest was found later, some measurements were re-calculated so that they reflected the situation on the day following clutch completion. This was possible because changes in the rate of reed growth and water level were monitored.

We assumed that even a small animal could reach reed warbler nests. Therefore, the nest survival should be primarily determined by the odds that it was detected and nests better concealed by the vegetation from above, from sides and from below, had a relatively higher chance of survival. "Concealment" may refer not only to visual, but also to acoustic and chemical cues – a thick layer of leaves and stems could block the transmission of stimuli more effectively than a thinner one [46]. Thus the nest site was described by three simple characteristics which were used as explanatory variables in models of survival:

- concealment from above – height of reeds measured above the nest rim (in centimetres)
- distance to a margin – distance to the nearest edge of the reedbed (a bay or open water of the pond; measured in meters on a scaled aerial photograph of the research plot)
- height – measured from the bottom of the pond to the bottom of the nest (in centimetres).

Vegetation changed over the season: newly growing reed stems which usually appeared in April, reached about 3.8 m by July. We therefore decided to use the date of laying of the 1st egg (expressed as the number of days that elapsed from the 1st May in the given year) as a covariate in all models.

### Ethics statement

All procedures regarding this field study were conducted according to the respective legislation of Poland. The permits to conduct the study involving a protected species in the protected area of nature reserve were issued by the Regionalna Dyrekcja Ochrony Środowiska (Regional Directorate of Nature Protection) in Wroclaw, Poland, and ringing licenses by the Ornithological Station of the Polish Academy of Sciences based on the decisions of Polish Ministry of Environment.

### Statistical analysis

Nest predation risk was analyzed using the Cox proportional hazard regression, a method which was originally developed for clinical studies, but has also been used in research of nest predation [5], [47]. Cox PH regression is a time-to-event analysis in which the survival time may be regressed against several independent variables and their interactions. This allows to predict a risk score for each nest. PH models can use all available information, including "incomplete" observations, like nests that were found some time after the clutch initiation or nests with unknown outcome (see below).

We could only test a general hypothesis that nest-site characteristics influenced nest predation risk. More explicit models testing direct mechanisms of predation, could not be constructed, because we did not know identities of predators and details of their searching tactics. For each breeding season we sought for the most parsimonious set of predictors of survival time (chosen from nest-site characteristics, the date of clutch initiation and their two-way interactions) and the final model was selected using the Akaike Information Criterion. Such an approach to modelling has serious limitations [48]. In particular, the final model may be used for prediction, but should not be used for testing hypotheses about statistical relationships between variables. We therefore could not conclude that selection pressures had differed between seasons, even if sets of independent variables in respective Cox PH models were unique in each year. Thus, we compared breeding seasons by a crossvalidation of hazards predicted for the same set of nests by models from different years. If mechanisms of selection were consistent across seasons, we should expect no significant differences between subsequent estimations of the hazard to the same set of nests.

All analyses were done with the R software (version 2.14; [49]). For the Cox PH regression models, we used the function ‘coxph' [50], with defined start/end times [51] and ties handled via Efron's method. Nests unsuccessful due to other reasons than predation (e.g. wind, death of nestlings) were included in the analysis. However, we right-censored the time of observation so that it did not consist of the day when the nest failed. A similar procedure was applied to nests which were parasitised by the cuckoo – the day preceding the day when the cuckoo's egg was laid, was set as the last day of the observation time. If such right-censored nests differed in a systematic way from other records with known outcome (fledged or depredated), this could bias results of PH models. Therefore, we compared characteristics of nest-sites in both groups. In five breeding seasons logistic regression models revealed no significant differences (likelihood ratio tests: 7.45> chi-squared>0.76, df  = 3, 0.860<P<0.059) and only in 2010 nests parasitised by cuckoos tended to be closer to the margin of the reedbed than remaining nests (chi-squared  = 10.68, df  = 3, P = 0.014). However, two Cox PH models for the 2010 season, with and without right-censored nests, produced almost the same results (respective values of R^2^ equalled 0.10 v. 0.09 and coefficients of concordance: 0.64 v. 0.64), which confirmed that right-censoring of a few observations could not bias results of the survival analysis.

All Cox PH models were checked for satisfying the proportional hazard assumption [52]. Hazard ratios were calculated using the function ‘predict' [50]. In an analysis of the effects of females' experience on the vulnerability of nests, we calculated hazard ratios using a script written in R language based on the hazard ratio formula [52].

## Results

Out of 524 nests, 244 (46.6%) fledged young and 280 (53.4%) were unsuccessful. Of failed 77% was due to predation (n = 215), and 23% (n = 65) for other reasons (wind or other adverse weather conditions, faulty nest construction, desertion by adult birds, death of nestlings, cuckoo parasitism). Most nest failure was due to predation (observed frequencies: 215 *v.* 65, expected: 140 *v.* 140; Χ^2^_1_ = 80.36, p<0.001). In all subsequent analyses, we focus only on the nest predation. The effect of other causes of nest failure was removed by right-censoring observation times of nests (see Methods).

**Figure 1:**
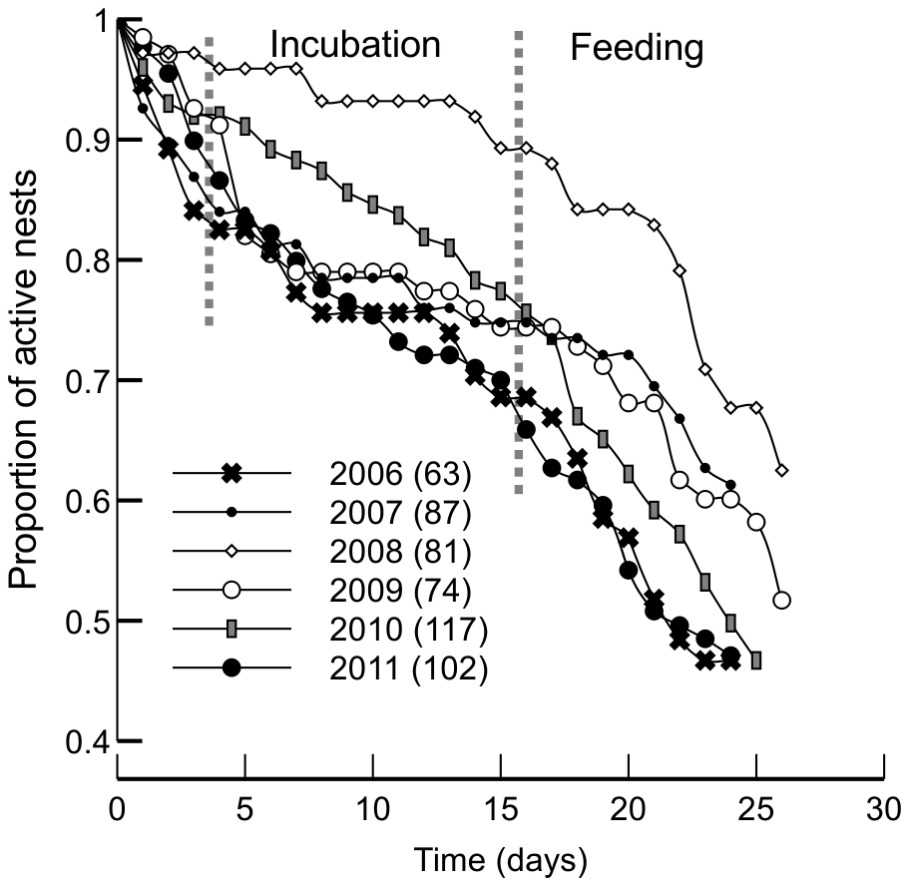
Kaplan-Meier survival curves for nests in 2006–2011 breeding seasons. Number of nests included in the analysis is given in parentheses next to seasons' labels. Day 1 is the day of laying of the 1st egg. There was a significant variation between seasons (log-rank test: Χ^2^_5_ = 12.6, p = 0.027).

In four seasons the mortality of nests due to predation was the highest at the egg-laying stage and the early phase of incubation (Fig. 1, Table 1). Thereafter, mortality stabilized at a lower level and finally accelerated again at the nestling stage. Two breeding seasons revealed different patterns, however (Fig. 1, Table 1). In 2010, the mortality rate appeared to be constant across the entire breeding cycle. In 2008 the survival curve was concave-up: mortality rate was rather low at the egg-laying and incubation stages and rapidly increased thereafter. Significant differences between some survival curves were found (see the legend to **Fig. 1**).

**Table 1:** Daily survival rates (expressed as percentages ± SE; numbers of nest-days given in brackets) of reed warbler nests at three stages of the breeding cycle.

Year	Egg-laying	Incubation	Feeding
2006	94.41±1.811 (170)	96.97±0.603 (502)	94.44±1.124 (298)
2007	95.36±1.510 (203)	98.77±0.408 (739)	97.95±0.641 (488)
2008	99.07±0.655 (217)	99.50±0.250 (803)	96.92±0.697 (635)
2009	97.51±1.099 (206)	98.11±0.540 (647)	97.25±0.784 (448)
2010	97.26±0.955 (300)	98.59±0.351 (1149)	95.30±0.838 (668)
2011	96.53±1.337 (269)	97.89±0.492 (872)	95.58±0.943 (496)

### Nest predation risk and nest characteristics

**Figure 2:**
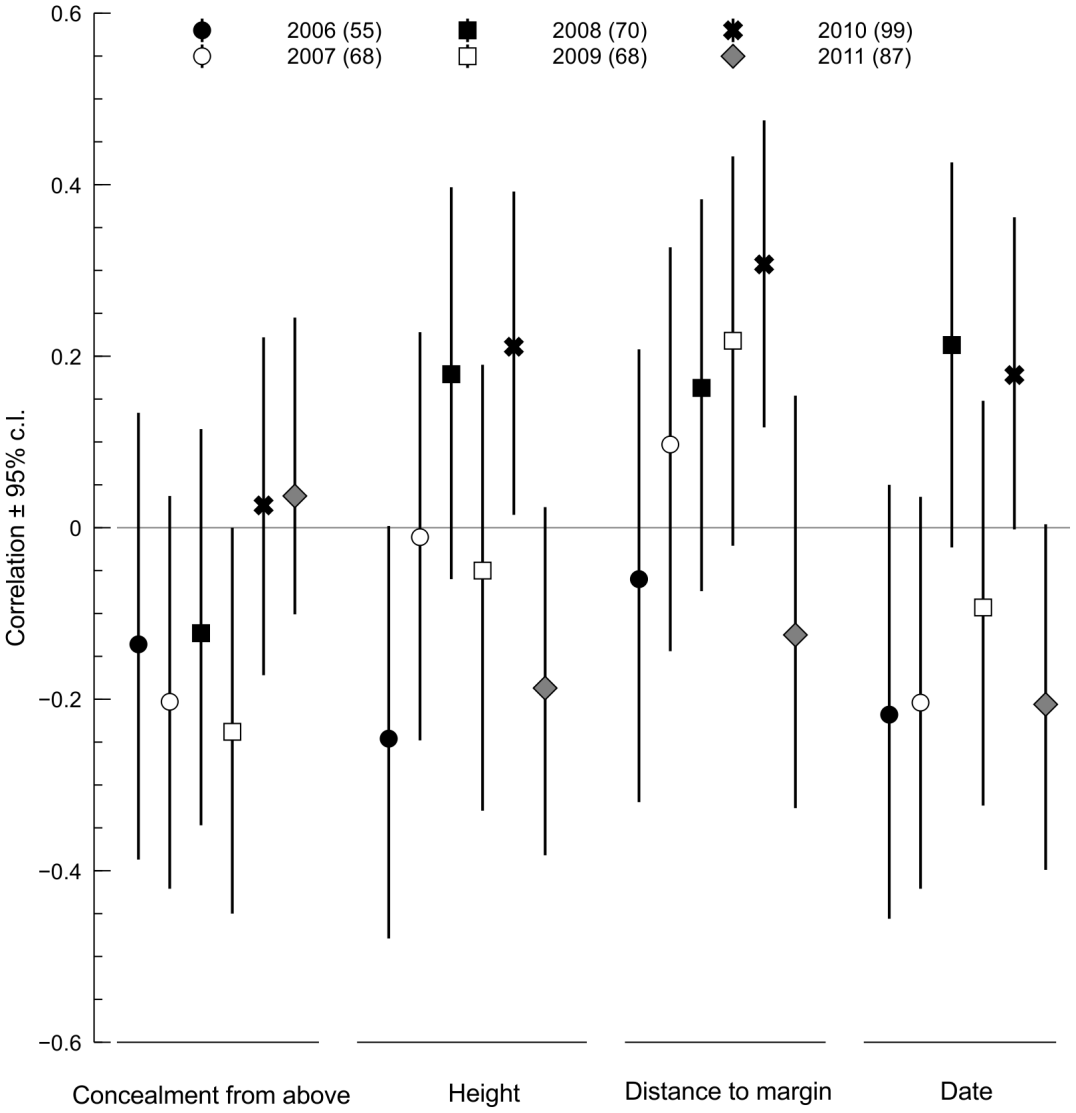
Point biserial correlations between nest-site characteristics and nests' fates. Positive coefficients indicate that relatively higher values of the trait were correlated with the higher incidence of nest predation. Only the nests found at the egg-laying stage were included.

**Fig. 2** presents the results of point biserial correlation analysis of relationships between characteristics of nests and their fates (depredated/successful). It appears that the same trait might covary with the nest fate in a different way in different seasons. Two characteristics, nest height and date of clutch initiation, were positively correlated with the predation risk in some years and negatively in others.

**Figure 3:**
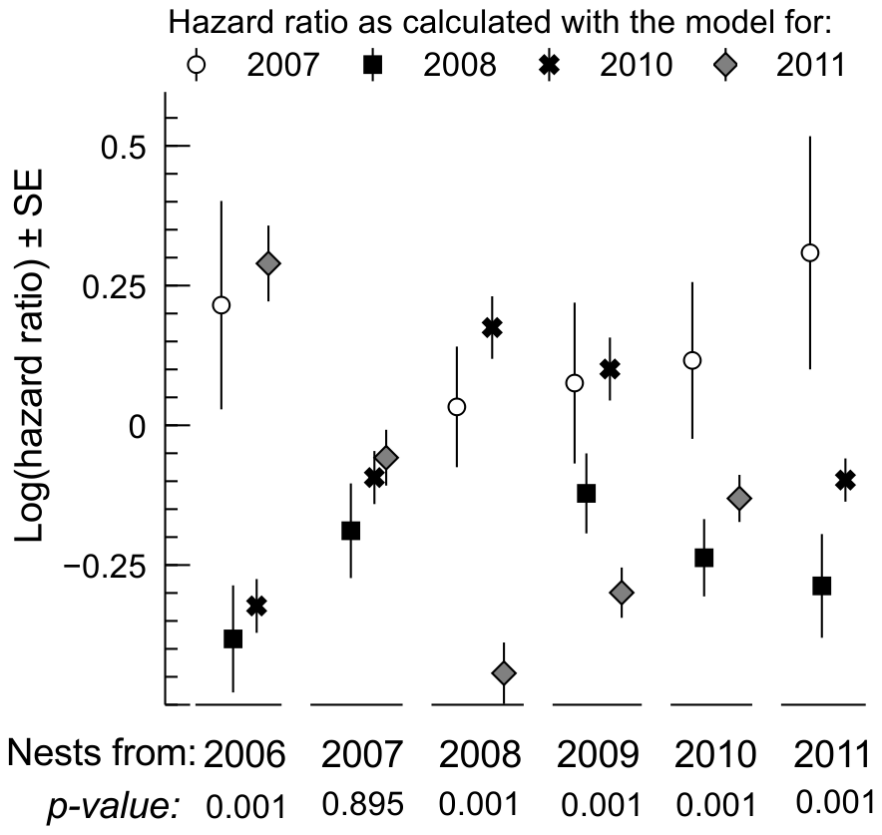
Crossvalidation of hazard ratios calculated for the same cohorts of nests with Cox PH models for different years. The logarithm of hazard ratio is grater than zero when a nest has greater chance of survival than a hypothetical nest with average values of predictor variables in the season for which the model was fitted, and lower than zero in the opposite case. P-values are Bonferroni-corrected and refer to Friedman nonparametric ANOVA. See **Figure 1** for sample sizes.

Cox PH models of nest survival were constructed separately for each season (see **S1 Table**). For 2006 and 2009, the received models had a poor predictive power. The models for the remaining seasons explained from 8 to 19% of variation in survival time and their predictive power was satisfactory (**Table 2**). To test the hypothesis that selective forces from nest predation differed between breeding seasons, we compared hazard predicted by different Cox PH models for the same set of nests (**Fig. 3**). Only in 2007 were average estimates of hazard ratios similar, irrespective of which model was used. As a rule, however, mechanisms generating variation in vulnerability of nests were vastly different between seasons. In other words, one particular nest could be considered either safe or susceptible to predation, depending on the season.

**Table 2:** Cox PH models explaining nest survival in 2006–2011 breeding seasons.

Year (no. nests)	LR test	R^2^	Concordance (95% c.l.)
2006 (63)	LR_1_ = 3.46, p = 0.063	0.053	0.61 (0.50–0.72)
2007 (87)	LR_7_ = 18.37, p = 0.010	0.190	0.73 (0.62–0.84)
2008(81)	LR_2_ = 14.04, p = 0.001	0.159	0.68 (0.56–0.80)
2009 (74)	LR_3_ = 6.00, p = 0.112	0.078	0.61 (0.49–0.73)
2010 (117)	LR_2_ = 10.84, p = 0.004	0.088	0.63 (0.55–0.71)
2011 (102)	LR_1_ = 8.72, p = 0.003	0.082	0.62 (0.53–0.71)

The likelihood ratio test verifies the general hypothesis that variables in the model explain a significant proportion of variance in survival time. The concordance coefficient measures predictive power (the higher the value, the better; 0.5 and lower values indicate that the model's performance is not better than guessing).

### Individual experience of females

We had data for 121 individually marked females that had two breeding attempts within a season. Of these, 31% of females (n = 37) bred successfully at both attempts, 17% (n = 21) were successful with the first nest but not the second, 38% (n = 46) were successful with the second nest but not the first, and 14% (n = 17) were unsuccessful at both attempts.

**Figure 4:**
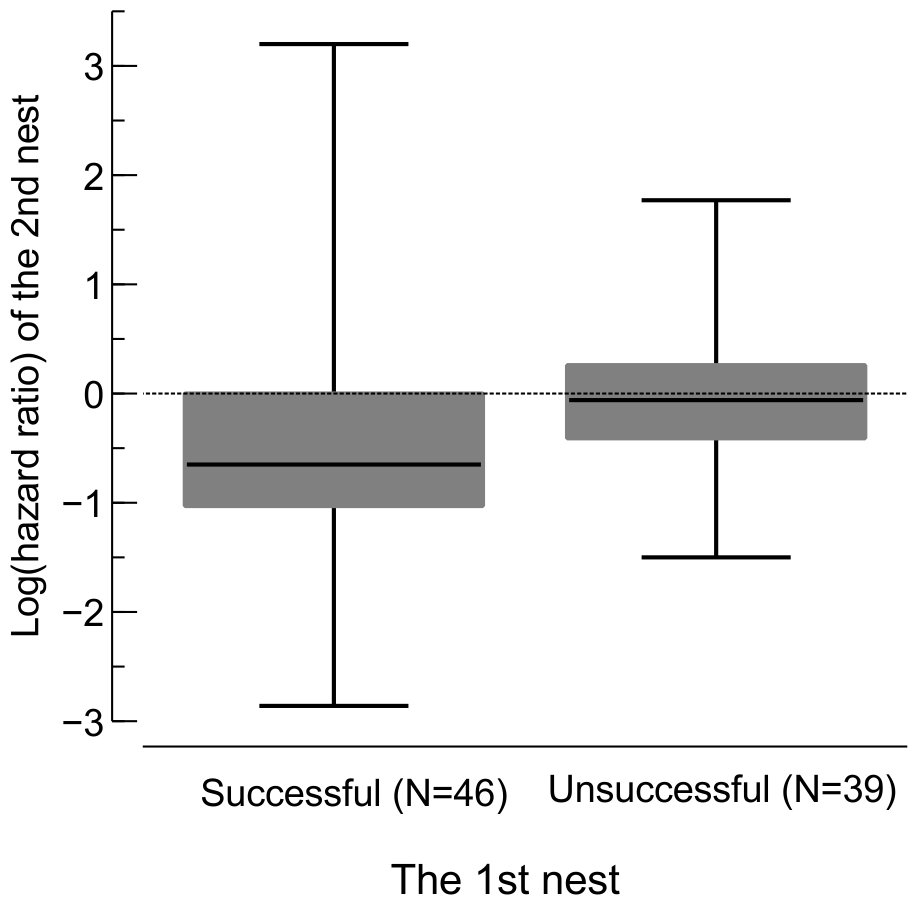
Log hazard ratios of the 2nd nest relatively to the 1st nest of the same female in the same breeding season. Medians, quartiles and ranges are shown. Horizontal dashed line indicates log hazard ratio equal 0 (both nests similarly vulnerable to predation). Females whose first nest was depredated had the second nest relatively safer than females successful at their first breeding attempt (t test: t_83_ = −2.71, p = 0.008; data from 2006 and 2009 were not included).

The distance females moved from the first to the second nesting attempt was independent of success (Mann-Whitney-Wilcoxon test: W = 1753, p = 0.702). To test the hypothesis that females after the failure of their first nest introduced adaptive changes in characteristics of their subsequent nest sites, we calculated hazard ratios for each second nest of the given female using Cox PH model for the corresponding breeding season (**Table 2**; females from 2006 and 2009 were excluded from the analysis because the corresponding Cox PH models had poor predictive power). Hazard to the female's first nest was used as a benchmark (technically speaking it was substituted into the numerator of the hazard ratio formula) for the hazard of her second nest. It appeared that females unsuccessful at the first breeding attempt in the given season, when compared to successful females, did significantly better at their second breeding episode (**Fig. 4**). This significant difference was not due to an improvement in choice of nest sites by females that failed at the first attempt (test of the null hypothesis that average log hazard ratio was equal zero, i.e. both nest sites were at similar hazard: one sample t-test: t_38_ = −0.14, p = 0.891), but rather due to a significant decline in the performance of previously successful females (t_45_ = −3.49, p = 0.001). In a Cox PH model stratified for season, the fate of the first nest predicted fate of the second nest (coefficient = −0.744, SE = 0.384; likelihood ratio test: LR = 3.91, DF = 1, p = 0.048) indicating a "protective effect" of the 1st nest failure. Overall, the females that experienced nest loss had the hazard to the subsequent nest reduced by twice (1/*exp*(−0.744) = 2.1) compared to the females that previously fledged some offspring.

**Figure 5:**
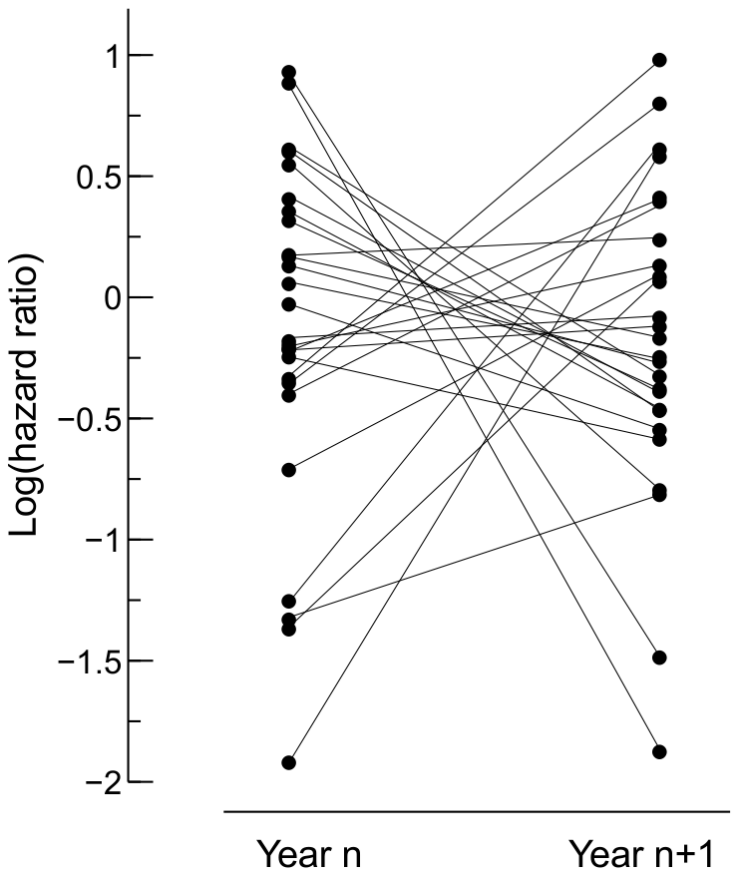
Hazard ratios of the 1st nests of 26 females in two subsequent breeding seasons. Points representing nests of the same female are connected with lines (paired-samples exact Wilcoxon test: V = 175, p = 0.99). Hazard ratio of the nest was calculated in relation to average values of nest site characteristics in the given season, using a respective Cox PH models presented in **Table 2** (data from 2006 and 2009 were not included).

It might be proposed that experience regarding nest placement accumulates with age. Thus females should build their nests in safer places in subsequent years. To test this, we compared the hazard of first nests (in the given breeding season) of 26 females in two consecutive years. The analysis revealed that 12 of them did better at the start of a new season and 14 did worse (**Fig. 5**). Thus, there was no tendency for an improvement and females generally seemed to start each season as novices.

## Discussion

Our population of European reed warblers, with 55% of nests unsuccessful and predators responsible for about 75% of all failures, is quite similar to other populations of small passerines [1]. Figures presented by Schulze-Hagen and co-workers [53], who summarized the results of 20 studies of reed warblers, were almost identical (on average 55.1% of nests failed and 71% of losses were due to predation) to our results. Only one study [45] produced different estimates: 45% failed nests and only 55% of all failures caused by predators. It should be noted, however, that these statistics were based on data from only one breeding season so they may not represent the average value for this population.

Perhaps the most important result from our study is a huge variability in breeding success and in models explaining nest survival. The extent of this is quite surprising considering that reedbeds are regarded as a rather simple and homogeneous habitat. Even so, the risk of predation and its characteristics changed quite dramatically. First, the rate of nest predation varied significantly between seasons and nest survival curves had different shapes (**Fig. 1**). Thus, depending on the season, relative vulnerability of nests in consecutive stages of the breeding cycle varied (for example, egg laying and early incubation was a vulnerable phase in 2006 and quite resistant to predation in 2008). Second, it appeared that selective pressures connecting nest-site characteristics to nest predation risk were unique for each year (**Figs. 2** and **3**).

Variability in the shapes of nest survival curves (**Fig. 1**) may reflect between-season changes in the proportion of different species in the guild of nest predators. Previous research has suggested that different predators tend to specialize in plundering nests at some particular stages of the breeding cycle [9], [32], [36], [54], [55]. Instability of proportions of predator species in the local guild might also be a factor responsible for between-season shifts of refuges. Our results suggest that safe nest sites were unrepeatable. With a cross-estimation of the predicted hazard of the same set of nest sites with Cox PH models from different years (**Fig. 3**), we showed that between-season shifts of refuges were real and should not be interpreted as a by-product of the modelling approach taken (selecting the most parsimonious set of predictors).

In summary, nest predation refuges of reed warblers do exist but they are not stable in time. How to breed in such a habitat? It seems that females did not use the ‘win-stay, lose-shift' strategy with regard to dispersal between breeding attempts within the same season [22], [23], [24], [25]. It appeared, however, that to some extent birds could manage the risk of the nest predation using experience gathered at the first breeding attempt in the given season [16], [56]. ‘Updating' of nest sites by females that failed at the first breeding attempt resulted only in maintaining the risk on the same level, so that the average log hazard ratio of nests was close to zero (**Fig. 4**). In contrast, females that were successful at the first breeding attempt tended to be conservative and copied their previous nest sites. As a result, since the habitat had already changed, the hazard to their second nests was significantly higher. Therefore, the females that were unsuccessful at the first breeding attempt, comparing to successful ones, had a significantly higher chance to fledge offspring in the subsequent episode. Thus, introducing adaptive changes allows only for keeping pace with the changing environment rather than gaining a substantial reproductive advantage, and staying idle results in a decline in performance.

An alternative explanation for a decline in nest mortality in the breeding episode following the failure of the 1st nest, might be that females did better because it was a kind of terminal investment [57]. Overwinter survival in small passerines is generally poor, thus the second breeding attempt in the current season might actually be the last one for many of birds [58]. Investment in nest defence can increase breeding success and even compensate for the risk connected to a vulnerable nest site [59], [60], [61]. It could therefore be proposed that parent birds, after the failure of their first nest, increased nest guarding at the subsequent breeding attempt, which resulted in an increase in the probability of success. Such a hypothesis, however, cannot account for the fact that a decline in hazard to the second nest of females that lost their first brood was the result in a model that included only characteristics of nest location, without any additional covariates connected to parental behaviour. Thus we would rather conclude that the observed decline in mortality was a result of adaptive changes in the nest position, even though some synergistic effects of changes in parental behaviour could not be ruled out.

Females were able to improve the safety of sequential nests within one season but they apparently could not use their experience in the subsequent year and started each season as naive individuals. This should not be explained as a general deficit of the cognitive system in a short-lived passerine, because small birds can collect relevant information and process it to position their nests progressively better as they age [20]. A more likely explanation is that in our study species the information collected in one year is simply not useful in the next season, because the environment has already significantly changed. Thus the results of analysis of longitudinal performance of individuals (**Fig. 5**) are in line with results demonstrating between-season shifts in nest predation refuges (**Figs. 2** and **3**).

To conclude, this work provides evidence that a risk to a nest is predicted by its characteristics, and documents a considerable temporal variation in nest predation risk. Nest predation refuges (safe nest sites) were predictable in short time-scale (within a season) but changed considerably between seasons: a nest safe in one season could have been vulnerable in another. Females could adapt to changing refuges: they used their experience from previous breeding attempts to cope with the changing environment. However, this strategy was effective only within one season and, because refuges shifted significantly between years, females could not accumulate experience to capitalize on ageing. An obvious next step is to determine whether the nest predation pattern is stable in space (for example in several adjoining habitat patches in the same breeding season). Finally, we should also ask how natural selection may operate if proximate mechanisms of predation are volatile.

## Supporting Information

S1 Table Cox PH models of nest survival.
(PDF)
