# Supplementary material for: Coping with Shifting Nest Predation Refuges by European Reed Warblers Acrocephalus scirpaceus
Source: PLoS One. 2014 Dec 18;9(12):e115456. doi: 10.1371/journal.pone.0115456 (PMC4270783; doi:10.1371/journal.pone.0115456)
Supplement: S1 Table — Cox PH models of nest survival. (PDF) [file pone.0115456.s001.pdf]

Table S1: Cox PH regression models explaining nest survival in 2006 – 2011 breeding seasons. Coefficients in the model equation, their standard errors and statistics of the Wald test are reported.

An initial model of nest survival for each season included the three nest site characteristics described in Methods, the date of clutch initiation and all pairs of two-way interactions. Thereafter the model was gradually reduced with the backward elimination method (one variable or interaction in each step), until the most parsimonious set of explanatory terms was received. We used Akaike Information Criterion values to select the best set of explanatory terms. Some terms were kept in the final model, if it was necessary to fulfil the principle of marginality.

Year (number of nests) and survival predictors	Coefficient	SE	z	p
**2006** (63)				
Date	-0.019	0.011	-1.82	0.070
**2007** (87)				
Concealment from above	-0.040	0.014	-2.88	0.004
Date	0.024	0.028	0.88	0.380
Distance to margin	-0.592	0.215	-2.75	0.006
Height	-0.018	0.018	-0.98	0.326
Distance to margin x concealment from above	0.003	0.001	3.11	0.002
Distance to margin x date	-0.004	0.002	-2.05	0.040
Distance to margin x height	0.002	0.001	2.08	0.037
**2008** (81)				
Concealment from above	-0.023	0.009	-2.58	0.010
Date	0.045	0.013	3.54	0.000
**2009** (74)				
Height	-0.049	0.022	-2.25	0.025
Date	-0.139	0.056	-2.50	0.013

Date x height	0.001	0.001	2.54	0.011
**2010** (117)				
Distance to margin	0.023	0.008	2.83	0.005
Date	0.019	0.008	2.38	0.017
**2011** (102)				
Height	-0.017	0.006	-2.91	0.004
